# Editorial: Antibodies and other biopharmaceuticals

**DOI:** 10.1002/biot.200800251

**Published:** 2008-10

**Authors:** Alois Jungbauer

Biopharmaceuticals are not any longer in their infancy, since their uprise in the nineties they are grown ups now. The general public and even scientists working in biological disciplines may not realize that in the Western world at least 20% of pharma-ceuticals are already of biological origin. What was a big innovation 30 years ago [1] has become reality [2]. When questioning why we have already 20–30% of biopharmaceuticals a simple answer is: they are efficacious, potent and safe. Only one of all approved biopharmaceutical needed to be removed from the market and biopharmaceutical companies thrive on their patents [3]. Biopharmaceuticals meet the therapeutic needs.

However, despite the clinical success of biopharmaceuticals, our society is facing a dilemma. Biopharmaceuticals may blow up the healthcare budget, as some of these drugs cost up to 35 000 US $ per therapy. So the social need to restrict health care expense is in conflict with the success of these new products.

The contribution of biotechnology will be to optimize production systems and even create new processes. This will not happen instantaneously, since processes are in place and a safe replacement is slow and requests additional clinical trials. Therefore, we will observe an incremental change within the next years. Eventually for new products we may observe a radical change in production technologies, using unit operations which have been around since a long time but will be refined for the needs of bio-pharmaceutical production. Re-combinant antibodies may be the first candidates for which we will see the implementation of these technologies.


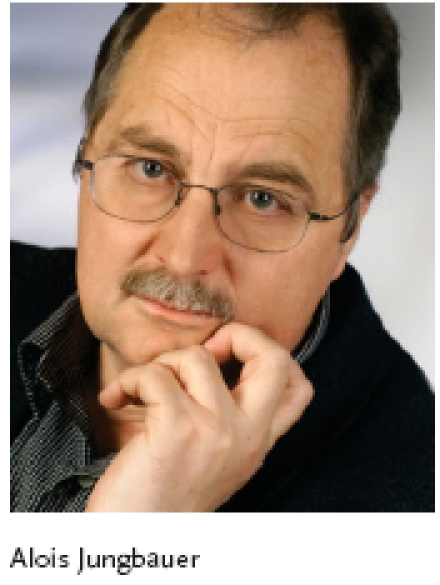


In the area of antibody manufacturing we also have observed a massive improvement of cell line productivity. They have been boosted to up to 10g/L and downstream processing cannot keep pace with this development. Although in downstream processing the constant improvement of chromatography material and membrane filters enable now productivities, which would have been unthinkable just a decade ago.

Whereas *Biotechnology Journal* has often highlighted the rise of biopharmaceuticals (for example in the special issues on biotech in China, Russia and Singapore [4–6]), several aspects of antibodies as therapeutics are addressed in this issue of *BTJ*. Ruuls *et al.* review a new class of antibody therapeutics: the age of the Umabs [7]. Zhou *et al.* review modern production technologies [8] and Garidel *et al.* report on a rapid, sensitive and economical assessment of monoclonal antibody conformational stability by intrinsic tryptophan fluorescence spectroscopy [9]. These three papers are a good attempt to get quick insights into the challenge of antibody processing.

## Direct links to references:

1 ↗ http://www.ualberta.ca/~csps/JPPS1(2)/biotech.htm

2 ↗ http://dx.doi.org/10.1038/nbt0708-743

3 ↗ http://dx.doi.org/10.1038/nbt0708-763

4 ↗ http://doi.wiley.com/10.1002/biot.200600083

6 ↗ http://doi.wiley.com/10.1002/biot.200790064

7 ↗ http://doi.wiley.com/10.1002/biot.200800110

8 ↗ http://doi.wiley.com/10.1002/biot.200800117

9 ↗ http://doi.wiley.com/10.1002/biot.200800091
